# Classical cuts: a pilot study of classical music’s effects on dogs in grooming settings

**DOI:** 10.1007/s10071-025-01984-9

**Published:** 2025-07-23

**Authors:** Wanda Krupa, Piotr Czyżowski, Kamila Kaszycka, Mirosław Karpiński, Joanna Liszka

**Affiliations:** Department of Animal Ethology and Wildlife Management, University of Life Sciences in Lublin, Lublin, Poland

**Keywords:** Dog grooming, Stress, Music, Welfare

## Abstract

Grooming procedures are often stressful for dogs due to exposure to loud noises, unfamiliar individuals, and the absence of their owners. This study aimed to assess whether classical music could reduce stress-related behaviours in dogs during grooming. Fifteen companion dogs of various breeds, aged 2 to 8 years, were observed during three grooming sessions: a control session without music, and two experimental sessions featuring classical piano compositions–Beethoven’s Moonlight Sonata and Chopin’s Nocturne. Music was played at 75 dB to mask ambient salon noise. Stress-related behaviours were rated on a 5-point scale during bathing, drying, clipping, and nail trimming. Results showed that all dogs, but especially males, exhibited significantly calmer behaviour in the music conditions. Female dogs showed similar trends, though differences were not statistically significant between stages. These findings suggest that classical music is a simple, effective, non-invasive enrichment method that can enhance dog welfare in grooming environments.

## Introduction

Grooming procedures can be highly stressful for dogs. During grooming, animals are exposed to various aversive stimuli, including unfamiliar people, the absence of their owners, lingering scents or the presence of other dogs, and loud noises. These stressors often trigger anxiety-related behaviours. While habituation can reduce stress responses over time, not all dogs adapt easily. Consequently, groomers increasingly seek effective, low-cost strategies to improve canine welfare during grooming.

One promising approach is auditory enrichment through music. This method is inexpensive and easy to implement. The influence of music on animal welfare depends on several factors, including sound frequency, volume, duration, species, and individual history of exposure (Castelhano-Carlos and Baumans 2009). In humans, music genres can differentially affect behaviour– classical music is typically calming, while fast rock music is arousing (Dalton and Behm 2007). Music’s analgesic and anti-anxiety effects are well-documented in humans and may be relevant for non-human animals despite perceptual differences (McCaffrey and Good 2000).

Stress in dogs can be assessed using physiological measures and behavioural observations. Behavioural indicators involve vocalisation, panting, lowered posture, paw lifting, body shaking, and stereotypic behaviours (Beerda et al. 2000).

Research suggests that music can improve the welfare of various animal species by masking ambient noise and reducing stress (Patterson-Kane and Farnworth 2006). In dogs, classical music has been shown to reduce vocalisations and tremors, while increasing restfulness. In contrast, heavy metal music may exacerbate stress responses (Kogan et al. 2012).

This study aims to investigate whether classical music can serve as an effective, non-invasive enrichment tool to reduce stress in dogs during grooming and enhance their overall grooming experience. Based on previous research, which demonstrated that auditory stimuli, especially classical music, can have calming effects on animals by reducing signs of stress (Bowman et al. 2015; Wells et al. 2002), it was hypothesised that exposure to classical music during grooming procedures would result in milder stress-related reactions in dogs compared to a silent control condition.

## Materials and methods

The behaviour of dogs during grooming procedures in a grooming salon was evaluated. The study included dogs that underwent grooming every 2–3 months: 15 adult dogs of various breeds aged 2 to 8 years; 10 males and 5 females. The dogs were groomed by the same groomer every session. The dogs’ behaviour was assessed during bathing, drying, clipping, and nail trimming using a 1–5 point scale (1 point– the dog shows strong signs of stress; behaves in a way that significantly hinders the procedure; 5 points– the dog is completely calm and relaxed). The same scorer assessed all the dogs. Calm behaviour that does not hinder the procedure was scored higher.

Evaluations were conducted during a control session (0), in which the only sounds present were those emitted by the grooming equipment, without the use of additional sound stimuli. Two test sessions (S and N) were carried out during subsequent visits to the salon, where additional acoustic stimuli were introduced: Ludwig van Beethoven’s Piano Sonata No. 14 in C-sharp minor, Op. 27 No. 2 (“Moonlight Sonata”) during session S, and Frédéric Chopin’s Nocturne in E-flat major, Op. 9 No. 2 during session N. The sound level of both pieces was set at 75 dB. The music was played using a Philips BTM 2310 audio system. The recordings were looped. Sessions were conducted in a fixed order. The first session was control one (0), followed by the S and N sessions.

Because the distributions of the dependent variables significantly diverged from normality, nonparametric (rank) tests were employed to analyse the significance. Dogs’ behaviour was compared both as a whole group between the sessions, as well as divided based on their sex. The Kruskal-Wallis test was used to compare mean scores between groups. The description of the distributions was based on the measures of the position of the mean value. The multiple statistical analysis of the research results was done using the Statistica 13.1 package.

## Results

When comparing the total scores obtained for individual grooming procedures (Table 1), statistically significant differences were observed between the control group and the groups exposed to classical music (*p* < 0.001) for three procedures: drying, clipping, and nail trimming. In both music groups– those exposed to pieces by Beethoven and Chopin– higher scores were recorded, suggesting milder reactions from the dogs during the procedures in the presence of music. For the bathing procedure, differences were also observed between the experimental and control conditions; however, these differences did not reach statistical significance.

**Table 1:** The effect of music playback on dogs’ reactions during grooming procedures (mean/median; (Q25–Q75), Kruskal-Wallis ANOVA) (0- control session; S- Ludwig Van beethoven’s piano sonata; N- Frédéric chopin’s Nocturne)

Grooming procedures	0	S	*N*	*p*-value
Bathing	3.3/3.0 (2.0–4.0)	3.9/4.0 (3.0–5.0)	4.1/4.0 (3.0–5.0)	*0.0700*
Drying	2.5/2.0^A^ (2.0–3.0)	3.5/4.0^B^ (3.0–4.0)	3.6/4.0^B^ (3.0–4.0)	*0.0001*
Clipping	2.7/3.0^A^ (2.0–3.0)	3.7/4.0^B^ (3.0–4.0)	3.7/4.0^B^ (3.0–4.0)	*0.0006*
Nail trimming	3.0/3.0^A^ (2.0–4.0)	4.1/4.0^B^ (4.0–4.0)	4.1/4.0^B^ (4.0–5.0)	*0.0002*

^AB^ values marked with different letters differ significantly (*p* < 0.001; post hoc test).

When comparing the average number of points obtained by dogs based on sex (Table 2), it was found that females scored significantly higher across all grooming procedures. This result indicates a difference in reactions to grooming procedures depending on the sex of the dog.

**Table 2:** Mean score values for individual grooming procedures depending on the sex of the dog

Grooming procedures	Male	Female	t	*p*-value
Bathing	3.4	4.4	-3.602	*0.001*
Drying	3.0	3.7	-3.074	*0.004*
Clipping	3.1	4.0	-3.962	*0.000*
Nail trimming	3.5	4.3	-3.358	*0.002*

When evaluating the impact of music playback depending on sex, it was found that during bathing there were no significant differences in the reactions of male and female dogs between the experimental conditions (*p* > 0.05) (Table 3). However, during drying, clipping, and nail trimming, a significant effect of music was observed, particularly in male dogs. In these three procedures, the dogs’ stress responses were significantly lower in the condition with music compared to the conditions without music (*p* < 0.01), as confirmed by the letter markings (a ≠ b). In female dogs, statistical significance was also found for the overall model (*p* < 0.05) in the cases of drying and clipping. Still, post hoc tests did not reveal differences between individual conditions, which may be due to the small sample size and limited test power.

**Table 3:** The effect of music playback on dogs’ reactions during grooming procedures by sex (mean/median; (Q25–Q75), Kruskal-Wallis ANOVA) (0- control session; S- Ludwig Van beethoven’s piano sonata; N- Frédéric chopin’s Nocturne)

Grooming procedures	Sex	0	S	*N*	*p*-value
Bathing	Male (*n* = 10)	2.9/3.0 (2.0–4.0)	3.6/3.0 (3.0–4.0)	3.8/4.0 (3.0–4.0)	*0.0836*
	Female (*n* = 5)	4.0/4.0 (3.0–5.0)	4.6/5.0 (4.0–5.0)	4.6/5.0 (4.0–5.0)	*0.4735*
Drying	Male (*n* = 10)	2.2/2.0^a^ (2.0–3.0)	3.3/3.0^b^ (3.0–4.0)	3.4/3.0^b^ (3.0–4.0)	*0.0005*
	Female (*n* = 5)	3.0/3.0 (3.0–3.0)	4.0/4.0 (4.0–4.0)	4.0/4.0 (4.0–4.0)	*0.0067*
Clipping	Male (*n* = 10)	2.4/2.0^a^ (2.0–3.0)	3.4/3.0^b^ (3.0–4.0)	3.4/3.0^b^ (3.0–4.0)	*0.0011*
	Female (*n* = 5)	3.2/3.0 (3.0–4.0)	4.4/4.0 (4.0–5.0)	4.4/4.0 (4.0–5.0)	*0.0384*
Nail trimming	Male (*n* = 10)	2.7/3.0^a^ (2.0–3.0)	3.8/4.0^b^ (4.0–4.0)	3.9/4.0^b^ (4.0–4.0)	*0.0007*
	Female (*n* = 5)	2.9/3.0 (2.0–4.0)	3.6/3.0 (3.0–4.0)	3.8/4.0 (3.0–4.0)	*0.0836*

^ab^ values marked with different letters differ significantly (*p* < 0.05; post hoc test).

An additional analysis comparing the average score values between the two music conditions for each grooming procedure did not reveal any statistically significant differences.

## Discussion

Environmental enrichment has been increasingly studied to enhance the welfare of kenneled or confined dogs (Wells 2009). Auditory enrichment reduces stress, supports species-specific behaviours, and minimises abnormal behaviours. Wells et al. (2002) reported that dogs exposed to classical music had more resting, less standing, and reduced vocalisation than during other auditory stimuli, suggesting a calming effect. Bowman et al. (2015) confirmed that classical music can reduce behavioural and physiological stress responses in shelter dogs. Their findings also suggest sex-based differences, with males being more responsive to auditory enrichment. These results are consistent with the findings of this study.

Several factors should be considered when implementing auditory enrichment. Music must not contain extreme frequencies and should remain below 60 dB (Patterson-Kane and Farnworth 2006). In noisier environments, such as grooming salons, the volume may need to be increased to mask aversive sounds, as was done in the present study, to 75 dB. Dogs show elevated blood glucose levels in response to noise above 80 dB (Treptow 1966), indicating a stress reaction (Kuo et al. 2015).

Not all auditory stimuli have calming effects. Harsh or abrupt sounds may increase arousal or fear (Köster et al. 2019). Individual variability plays a significant role in auditory response. Hearing ability, which may decline with age or differ by breed, also influences effectiveness (Fefer et al. 2022).

This study selected Beethoven’s Moonlight Sonata in C-sharp minor and Chopin’s Nocturne in E-flat major due to their slow tempo, soft dynamics, and absence of vocals or abrupt changes. These piano pieces, with frequencies between 104 Hz and 1.3 kHz, fall within the auditory range of dogs and humans. No statistically significant differences were found between both pieces indicating, with tempos around 60–80 BPM, they may entrain biological rhythms and contribute to stress reduction.

This study had a few potential limitations. The first issue was that the order of the sessions was fixed, which could potentially impact the dogs’ behavioural responses. However, all of the dogs were regular customers at this specific groomer, so they were accustomed to the settings in that particular salon. The dogs’ behaviour did not differ significantly between any of the auditory enriched sessions; therefore, the order of the sessions is unlikely to have had a significant impact on the results of this study. Another limitation was the decibels at which the music was played. While the music needed to be heard and cover some of the sounds, it might have been too loud, therefore lowering the scores of some dogs. Despite that, the music was played at the volume typical for this workplace setup. Another limitation was that only one scorer was assessing the dogs’ behaviour during the session. This single evaluator has extensive experience as a professional groomer, having worked with these dogs over many sessions. This evaluator’s long-term familiarity with the individual animals and their typical behavioural responses during grooming allowed for consistent and informed assessments.

## Conclusion

Auditory enrichment by music effectively reduces stress-related behaviours in dogs across every phase of the grooming process. As only two musical pieces with similar characteristics were used, it is not possible to determine which specific elements of the music contributed to this effect. Further research using a broader range of musical styles and structures is needed to identify the key components that influence dogs’ stress responses. Because male dogs experienced a significantly greater stress reduction, sex‐specific differences in auditory sensitivity or stress physiology may need further investigation. These findings support the integration of playlists into grooming salons as a low‐cost means to promote dogs’ relaxation and improve their mood. Future research should broaden the investigation to include additional timbres, dynamics, and tempo variations. This work confirms that targeted auditory enrichment is a practical strategy for managing stress during canine grooming.

## Data Availability

No datasets were generated or analysed during the current study.
